# Mesenchymal Stem Cell-Derived Extracellular Vesicles for Corneal Wound Repair

**DOI:** 10.1155/2019/5738510

**Published:** 2019-12-09

**Authors:** Hongyan Tao, Xiaoniao Chen, Hongmei Cao, Lingyue Zheng, Qian Li, Kaiyue Zhang, Zhibo Han, Zhong-Chao Han, Zhikun Guo, Zongjin Li, Liqiang Wang

**Affiliations:** ^1^Nankai University School of Medicine, Tianjin, China; ^2^Department of Ophthalmology, Chinese PLA General Hospital, Beijing, China; ^3^School of Life Science and Technology, China Pharmaceutical University, China; ^4^Tianjin Key Laboratory of Engineering Technologies for Cell Pharmaceutical, National Engineering Research Center of Cell Products, AmCellGene Co., Ltd., Tianjin, China; ^5^Jiangxi Engineering Research Center for Stem Cell, Shangrao, Jiangxi, China; ^6^Henan Key Laboratory of Medical Tissue Regeneration, Xinxiang Medical University, Xinxiang, China; ^7^State Key Laboratory of Kidney Diseases, Chinese PLA General Hospital, Beijing, China

## Abstract

With the immunoregulation potential, mesenchymal stem cells (MSCs) have been used for tissue regeneration by relieving inflammation in the injured tissues. When this repair process is interfered by immune disorders or pathological angiogenesis, the delays in corneal epithelial wound healing can lead to a persistent epithelial defect. Stem cell-derived extracellular vesicles (EVs), which carry abundant bioactive molecules from stem cells, have provided an alternative to regeneration therapy. In this study, we aimed to investigate if EVs from human placenta-derived MSCs (hP-MSCs) could ameliorate alkali injury of the cornea in the mouse model. 33.33 *μ*g/*μ*L EVs in 10 *μ*L PBS were applied to the cornea. Repeat application three times, and 100 *μ*g EVs (in 30 *μ*L PBS) in total were administrated per day for two weeks. Our results revealed that EVs from hP-MSCs had preferable functions including enhancing proliferation and anti-inflammation and suppressing apoptosis of corneal epithelial cells. Furthermore, hP-MSC-derived EVs ameliorated mouse corneal wound healing by inhibiting angiogenesis and inflammation. Taken together, our current data suggested that hP-MSC-derived EVs have the beneficial effects of corneal wound healing, which provide alternative cell-free therapy with great practical value.

## 1. Background

Wound healing is a complex process involved in tissue regeneration and functional recovery [1]. Different from other tissues and organs, the cornea is avascular and the disruption of corneal transparency after injury could lead to major irreversible blindness thereby reducing the quality of life [2, 3]. The injuries, as well as a delay in wound repair, would disrupt corneal angiogenic privilege and trigger corneal neovascularization, which leads to a complete loss of vision. The repair of the damaged tissue including multiple cellular processes with frequent movement and proliferation of cells is the leading process [4]. Topical drug delivery with eye drop is the most accessible and noninvasive method. However, the normal ocular layer has impervious nature of the ocular surface due to tight epithelial junctions; constant blinking and tearing would rapidly clear the eye drops from the ocular surface in a few minutes [5]. Moreover, the blood capillaries in the conjunctival could absorb the delivered drug, which further reduces the amount of drug available for effective ocular absorption. Hence, development of a novel drug delivery system that can surmount the ocular surface barriers and release the drug for extended periods of time, thus enhancing therapeutic efficacy and improving patient compliance, is vitally important to treat ocular injuries and restore normal physiological functions of the eye.

Human placenta-derived mesenchymal stem cells (hP-MSCs) have greater proliferation and differentiation capabilities. Furthermore, the source of these cells is generally disposed as medical waste without ethical issues [6]. An increasing number of results have shown that MSCs possess therapeutic effects ranging from ameliorating acute and chronic liver damage and heart failure to wound healing [7, 8]. In addition, recent studies have shown that MSC therapies can attenuate optic nerve injury and enhance the retinal ganglion cells [9]. However, MSC transplantation inevitably faces many potential risks, such as ethical issues and immune responses. Compared with MSCs, MSC-derived EVs have a low propensity to trigger immune responses and a reduced risk of ectopic engraftment [10, 11]. Over the last few years, there have been advances that in addition to direct cell transplantation, the paracrine effect of MSCs is one of the mechanisms that play an important role in the treatment of diseases. Vast studies have shown that MSC-derived EVs contribute to the therapeutic potency of MSCs by transporting paracrine factors [12–14]. Thus, MSC-derived EVs can provide beneficial effects parallel to those of MSC transplantation [10, 15].

## 2. Materials and Methods

### 2.1. Cell Culture

Human placenta-derived MSCs (hP-MSCs) were harvested as reported previously [16] and cultured in complete Dulbecco's modified Eagle's medium (DMEM)/F12 (Gibco, Grand Island, NY) with 100 U/mL penicillin-streptomycin (P/S) (Gibco) and 10% fetal bovine serum (FBS; HyClone, Australia), bovine insulin (5 *μ*g/mL), and recombinant human epidermal growth factor (10 ng/mL) [3]. Exosome-free FBS was obtained from FBS centrifuged at 100,000 g for 70 min [17]. hP-MSCs between passages 7 and 10 were used for subsequent experiments. The human corneal epithelial cells (HCEs) were purchased from the ATCC and cultured as the instruction described.

### 2.2. Flow Cytometric Characterization of hP-MSCs

The hP-MSCs were dissociated with 0.25% trypsin-EDTA (Gibco, Grand Island, NY) and then washed with PBS containing 2% FBS. Afterwards, the cell suspensions were incubated with fluorescence-conjugated antibodies including CD44, CD90 (Abcam, Cambridge, MA), or unstained control for 30 min at room temperature. Then, the cells were washed with PBS and resuspended in FACS buffer. The FACS analysis was performed using a FACSCalibur™ flow cytometer (BD Biosciences), and the data were analyzed using the Cell Quest Pro software (BD Biosciences).

### 2.3. EV Isolation, Characterization

The method for EV isolation and characterization was performed as our previous and other groups' report [11, 18, 19]. The internalization of EVs was analyzed with the CM-DiI membrane dye (Invitrogen, Carlsbad, CA), according to our previous report [11].

### 2.4. Western Blot Analysis

HCEs were harvested in RIPA lysis buffer, quantified by using a BCA Protein Assay Kit (Promega), separated by 10% SDS-PAGE, and transferred onto polyvinylidene fluoride membranes (Millipore, Darmstadt, Germany). The membranes were blocked in 5% nonfat milk for 2 h and incubated in primary antibodies overnight at 4°C followed by incubation with horseradish peroxidase- (HRP-) conjugated secondary antibodies for 1 h at room temperature. Signals were visualized with an Immobilon™ Western Chemiluminescent Horseradish Peroxidase (HRP) substrate. GAPDH was used as an internal control. The following primary antibodies were used: CD9 (1 : 200, rabbit IgG), CD63 (1 : 200, rabbit IgG), and GAPDH (1 : 200, rabbit IgG).

### 2.5. Cell Proliferation and Cytotoxicity Assay

HCEs were seeded at 2 × 10^3^ cells/well into a 96-well plate in basal DMEM/F12. Extracellular vesicles (20 *μ*g/mL, 40 *μ*g/mL, 60 *μ*g/mL, 80 *μ*g/mL, and 100 *μ*g/mL) were added for 24 and 48 h. MTT solution was dissolved in 100 *μ*L of dimethyl sulfoxide (DMSO). The optical density was measured at 490 nm on a multiwell plate reader.

### 2.6. Ki67 Immunostaining

For cell proliferation assessment, HCEs were cultured in 100 *μ*g/mL extracellular vesicles for 12 and 24 h. After fixing in 4% paraformaldehyde for 15 min, the slides of cells were performed immunostaining with antibody against Ki67 (BD Pharmingen, San Jose, CA). And the nuclei were stained using 4,6-diamidino-2-phenylindole (DAPI, BD Biosciences) as counterstaining.

### 2.7. Scratch Wound Healing Assay

HCEs were seeded at 3 × 10^4^ in a 6-well plate; when they reached confluence, scratch wounds were generated across each well using a sterile plastic 20 *μ*L micropipette tip. After washing cells twice with PBS, DMEM/F12 containing EVs at a final concentration of 100 *μ*g/ml was added to cells. The migratory effect was quantified by measuring the residual fractional wound area using ImageJ software (NIH, https://imagej.nih.gov/ij/).

### 2.8. Real-Time PCR

Total RNA was isolated from cells or tissues. Total RNA was also harvested from cornea alkali injury tissue. Real-time PCR was performed on a CFX96TM Real-Time PCR System (Bio-Rad, Hercules, CA) using SYBR Green-based real-time detection method (Roche, Mannheim, Germany). The relative expression level of the mRNA of interest was expressed as 2^-*ΔΔ*CT^ and normalized to GAPDH, respectively. The primer sequences used in this study were listed in [Supplementary-material supplementary-material-1].

### 2.9. Animal Model and EV Therapy

An alkali burn injury model of the cornea in the mouse was performed as reported previously [20]. FVB mice were randomly divided into three groups (*n* = 8 for each group) postinjury, and the next day, 33.33 *μ*g/*μ*L EVs in 10 *μ*L PBS were applied to the cornea. PBS only was used as the control. Repeat application 3 times, and 100 *μ*g EVs were administrated per day for 2 weeks. Protocols were approved by the Nankai University Animal Care and Use Committee guideline, which conforms to the *Guide for the Care and Use of Laboratory Animals* published by the US National Institutes of Health (8th Edition, 2011).

### 2.10. Corneal Fluorescein Staining

Corneal fluorescein sodium staining was used to detect the epithelial repair process on day 7. After intraperitoneal anesthesia with 4% chloral hydrate in mice, the sodium fluorescein filter paper was dampened with sterile PBS, lightly attached to the mouse cornea, and then rinsed with PBS to remove excess fluorescein. Observe the damage of the cornea under ultraviolet light and take a photo with a digital camera.

### 2.11. Corneal Neovascularization Assessment

We used a stereomicroscope to observe the neovascularization of the eye in mice at 5 days after surgery. Perform a daily examination of the mice in a blinded fashion under a stereomicroscope. This assessment was performed everyday considering the growing speed of the neovascularization.

### 2.12. Histological Analysis

On day 7 or 14 post administration, all mice were sacrificed, and the corneas were excised. Some corneal tissues were immediately fixed in 4% PFA overnight. Afterwards, these tissues were embedded in paraffin, sectioned at 5 *μ*m, and then subjected to hematoxylin and eosin (H&E) trichrome staining. The slices obtained were examined with an optical microscope (OLYMPUS BX51, Japan). For corneal neovascularization analysis, animals were euthanized on day 14, and corneal tissue was embedded into OCT compound (Sakura Finetek, Japan). Samples were cut into 5 *μ*m thick sections for immunofluorescence staining. Cell nuclei were counterstained with DAPI. The sections were incubated with primary antibody against CD31 (rat anti-mouse, BD Biosciences) overnight at 4°C and then incubated with Alexa Fluor 594 goat anti-rat IgG (Invitrogen, Grand Island, NY). The newborn capillary vessels were observed using a fluorescence microscope (×200).

### 2.13. Statistical Analysis

All data were expressed as the means ± SEM. Significance of difference between two groups was tested by Student's *t*-test or ANOVA. Fisher's exact test (Freeman-Halton) was employed to assess the outcome of treatment. Differences were considered significant at *P* values < 0.05.

## 3. Results

### 3.1. Isolation and Characterization of EVs

**Figure 1:**
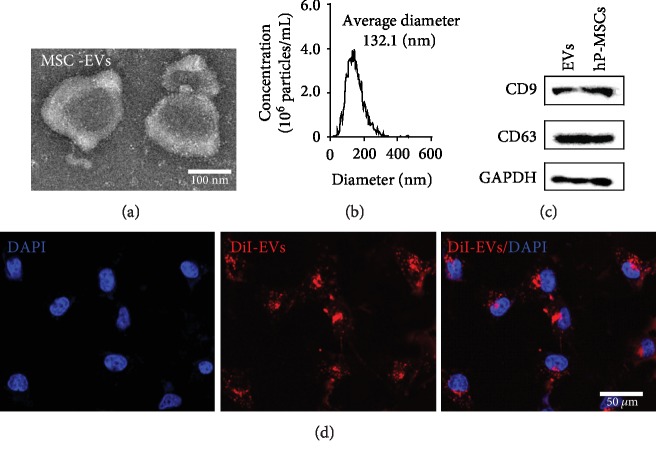
Characterization of EVs from hP-MSCs. (a) Representative transmission electron microscopy (TEM) image of isolated EVs. Scale bar, 50 nm. (b) The diameter of EVs was around 130 nm as showed by Nanoparticle Tracking Analysis (NTA). (c) Western blot results showed the expression of CD9 and CD63 in EVs and hP-MSCs. (d) The uptake of EVs (red) by HCEs. Scale bar, 50 *μ*m.

Prior to extracting EVs from medium conditioned by hP-MSCs, we determined the phenotypic properties of hP-MSCs by flow cytometry. As shown in [Supplementary-material supplementary-material-1], MSCs maintained the expression of surface markers CD90 and CD44. The hP-MSC-derived EVs were isolated as reported previously and subject to biochemical and biophysical analyses. TEM analysis showed that EVs exhibited cup-shaped morphology (Figure 1(a)). NTA revealed that the average diameter of EVs was approximately 130 nm (Figure 1(b)), and 99.4% of EVs were in the 132.1 nm ([Supplementary-material supplementary-material-1]). In addition, HCEs also release EVs with similar diameter ([Supplementary-material supplementary-material-1]). Western blot analysis of EVs showed a positive expression of the protein content and the EV proteins CD9 and CD63 (Figure 1(c)). To determine whether the EVs were internalized by HCEs, EVs were labeled with CM-DiI dye (red) and incubated with HCEs *in vitro*. After 24 h, the photomicrographs showed that the labeled EVs were taken up by HCEs (Figure 1(d)). The results demonstrated that the EVs were transferred into HCEs *in vitro*.

### 3.2. Enhanced Proliferation Effects of EVs *In Vitro*

**Figure 2:**
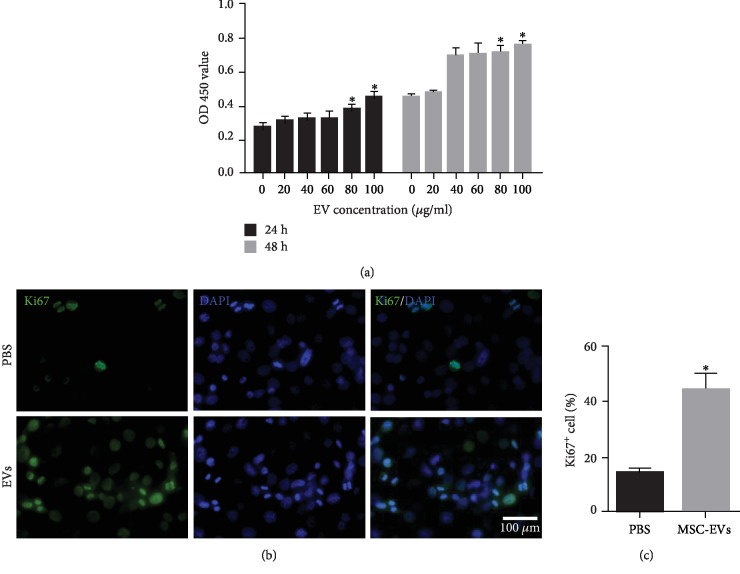
The proliferative effects of hMSC-derived EVs on HCEs. (a) MTT assay revealed the increased proliferation of HCEs under EV treatment with a dose-dependent manner (the concentration of EVs was increased from 0 to 100 *μ*g/mL, using PBS for dilution). (b, c) Ki-67 staining showed that the proliferation of HCEs was significantly improved after incubating with EVs and lasts for 48 h. All data is shown as means ± SEM in triplicate assays. ^∗^*P* < 0.05 versus PBS.

To analyze the optimal working concentration of EVs, HCEs were cultured with EVs for 24 and 48 h. An MTT assay showed that, compared with PBS, incubation with EVs could increase proliferation of HCEs after 24 and 48 h (Figure 2(a)). There appears to be a dose-dependent effect with increasing concentration of EVs. It revealed that EV concentration at 100 *μ*g/mL and cultured with hP-MSCs for 48 h was the most beneficial for the viability of HCEs. Hence, EVs have been suggested to play a crucial role in tissue regeneration by promoting cell proliferation. Ki-67 staining showed that the proliferation of HCEs after incubating with EVs was significantly improved (Figures 2(b) and 2(c)).

### 3.3. Promoted Corneal Wound Healing of EVs

**Figure 3:**
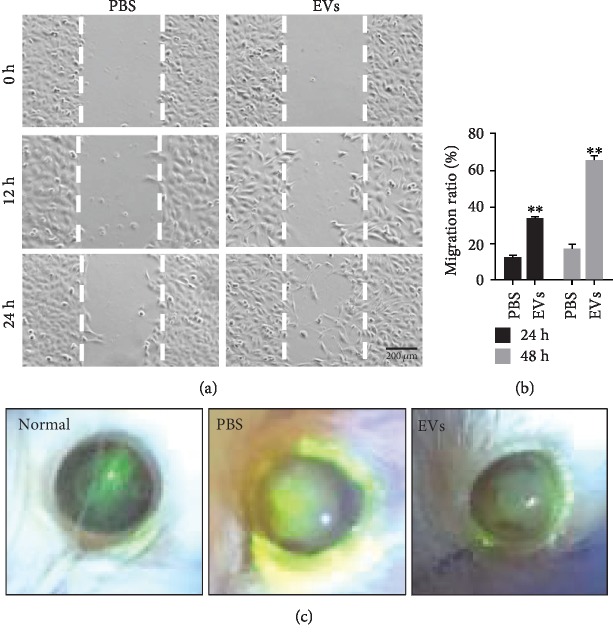
The therapeutic effects of EVs on corneal wound healing. (a, b) Wound healing assay revealed that EVs markedly increased the cell migration of HCEs. All data were shown as means ± SEM in triplicate assays. ^∗∗^*P* < 0.01 versus PBS. (c) Fluorescein-stained images showed the improved therapeutic effect of EVs on corneal wound healing *in vivo* (*n* = 8).

To detect the activities and promigratory effects of EVs released from hP-MSCs, we performed a scratch wound healing assay. The results showed that EVs released from hP-MSCs significantly promoted epithelial cell migration after incubation for 12 and 24 h (Figures 3(a) and 3(b)). We next examined whether EVs could promote corneal epithelial wound healing *in vivo*. Fluorescein stained images of 2 mm wounded mice corneas, before and 7 days after treatment with either hP-MSC-derived EVs or vehicle control. As shown in Figure 3(c), the EV-treated group had healed significantly more than the control group.

### 3.4. Inhibited Inflammation and Apoptosis of EVs

**Figure 4:**
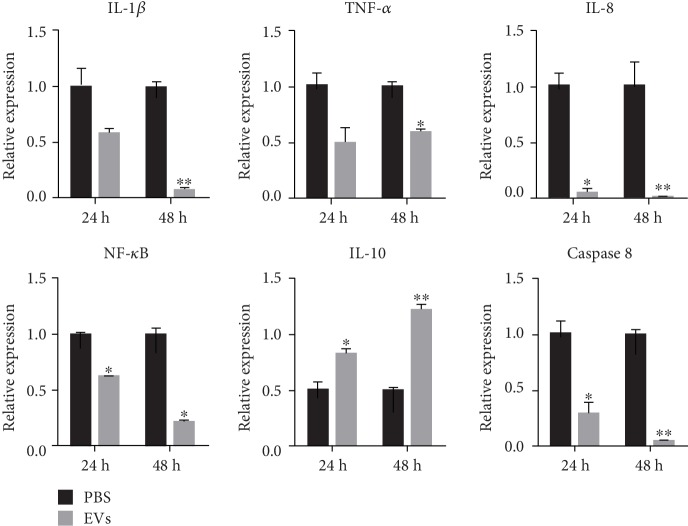
RNA expression level of the immune-related gene *in vitro*. qRT-PCR revealed that mRNA levels of IL-1*β*, IL-8, TNF-*α*, and NF-*κ*B in HCEs were highly decreased after treatment with EVs, while anti-inflammatory factor IL-10 was increased *in vitro*. In addition, the EV group significantly reduced apoptosis-related gene caspase 8 expression compared with the control groups. All data were shown as means ± SEM in triplicate assays. ^∗^*P* < 0.05, ^∗∗^*P* < 0.01 versus PBS.

Next, we examined whether EVs could inhibit tissue inflammation and apoptosis. The HCE apoptosis genes and inflammation-related factor genes were assessed by qRT-PCR analysis. The results revealed that RNA levels of IL-1*β*, IL-8, TNF-*α*, and NF-*κ*B were significantly decreased after treatment, both *in vivo* and *in vitro*, while anti-inflammatory factor IL-10 was increased *in vitro*. At the same time, the expression of apoptosis-related genes Cas-8 was decreased, indicating that EVs play a key role in inhibiting apoptosis (Figure 4).

### 3.5. EVs Regulated Angiogenesis of Cornea

**Figure 5:**
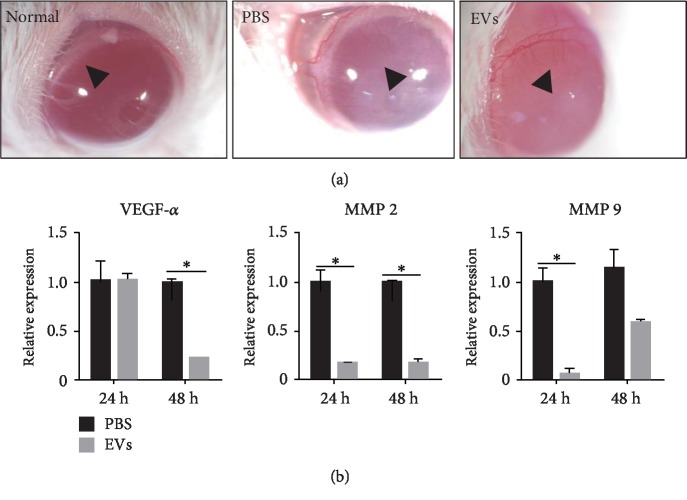
Inhibited cornea angiogenesis with EV treatment. (a) Injury-induced angiogenesis was significantly inhibited by EV applications (arrowhead). The arrowheads point out the margin of the corneal neovascularization. The distance of the margin of corneal neovascularization of EVs was longer than that of the control group and close to that of the normal group. In addition, in the PBS group, the opacification area was larger than that of the EVs group. The therapy improved the corneal transparency. (b) qRT-PCR revealed that angiogenesis-related mRNA levels of MMP2, MMP9, and VEGF-a were significantly decreased after treatment. All data were shown as means ± SEM in triplicate assays. ^∗^*P* < 0.05 versus PBS.

To determine the angiogenesis regulation potential of EVs, we used a stereomicroscope to observe the neovascularization of the injured eye in the following 5 days and took pictures at the fifth day after surgery both in the therapy group and the control group. As shown in Figure 5(a), the pathological angiogenesis in the treatment group was significantly reduced, and the ocular transparency of the EV group was better than that in the PBS group. Furthermore, we measured the expression of the proangiogenic genes, VEGF-a and angiogenesis-associated matrix metalloproteinases 2 (MMP2) and MMP9, highly decreased after treatment (Figure 5(b)). Immunostaining of CD31 at day 14 also revealed that microvascular density was significantly decreased by application of EVs released from hP-MSCs, which was consistent with the H&E results ([Supplementary-material supplementary-material-1]). All these data suggested that EVs regulated corneal pathological angiogenesis.

### 3.6. EVs Increased Tissue Repair in Alkali Burn Model

**Figure 6:**
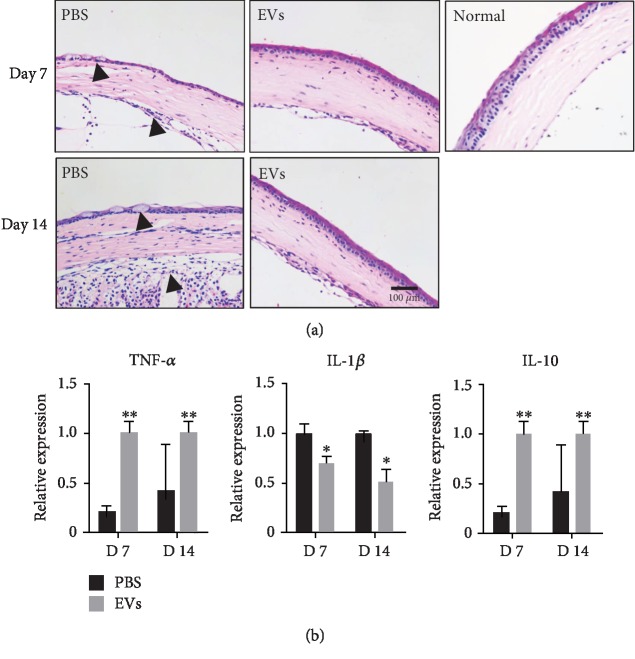
Enhanced therapeutic effect of EVs in the alkali burn model. (a) H&E staining of the cornea demonstrated stromal edema and inflammatory cell infiltration on days 7 and 14 after injury in EV-treated mice, compared with the cornea without injury. The arrowheads pointed to the disorganized fibers and phlyctenule in the epithelium layer. The degree of repair of the epithelial layer in the control group was significantly lower than that in the treatment group. (b) qRT-PCR revealed that mRNA levels of TNF-*α* and IL-1*β* were highly decreased after treatment, while anti-inflammatory factor and IL-10 were increased *in vivo*. All data were shown as means ± SEM in triplicate assays. ^∗^*P* < 0.05, ^∗∗^*P* < 0.01 versus PBS.

In order to elucidate the mechanism of corneal alkali damage repair capacity of EVs derived from hP-MSCs, the corneal alkali damage mouse model was used in this study. As shown in H&E staining, the normal corneal epithelium is composed of 4-5 layers of epithelial cells. Compared with the EV treatment group, the control group has a loose and disorder arrangement of the fibers of the inherent collagen layer, and a large number of cracks or vacuoles were observed between the fibers (Figure 6(a)). After 7 days treatment, the newborn epithelium is thinner and there are fewer stromal cells in the control group. On the other hand, the corneal epithelium of the EV-treated groups basically recovers 4-5 cell layers of structure, with relatively regular corneal matrix arrangement after treatment (Figure 6(a)). Tissue RNA was extracted in the EV group and control group. The qRT-PCR analysis of corneal tissues showed that in the treatment group, EVs can significantly reduce the expression of inflammation-related factor genes both in the early phase (24 h) ([Supplementary-material supplementary-material-1]) and during the therapy phase (Figure 6(b)). This suggested that the corneal epithelial cell layer recovered rapidly in the treatment group and formed a tight link with the corneal stroma.

## 4. Discussion

Corneal chemical injury is a common type of injury that often causes extensive damage or even permanent visual impairment. During the corneal repair process, modifying the local environment and stimulating local cell response after injury are essential for corneal tissue repair. In this study, our results revealed that EVs from hP-MSCs promoted mouse corneal wound healing by inhibiting angiogenesis and inflammation, which provide alternative cell-free therapy for translational application.

MSCs have been proved to possess a potential therapeutic value in autologous or allogeneic transplantation in the field of stem cell-based tissue repair [12, 13]. Among these studies, donor cells were detected to migrate to the injury site and suppress the activity of the immune cells. This process is mediated by the production of immunoregulatory factors and thereby facilitate tissue repair in response to the local inflammatory environment [21]. In this experiment, we did not employ the widely used bone marrow-derived MSCs but instead used placenta-derived MSCs. As one of the accessory products of the fetus, the placenta can provide a relatively abundant and harmless source of stem cells. The application of such cells increases the likelihood that the results of this experiment will be practically applied in the clinic [6].

In addition, many research suggested that the paracrine mechanisms of MSCs exert an important effect on the regulation of vascular remodeling [22] and improvement of organ recovery [23]. Since the important role of MSC paracrine effects in their therapeutic ability *in vivo*, we want to examine the physiological process of EVs secreted by MSCs in the injury area. Western blots confirmed the presence of EV marker proteins CD9 and CD63 [24], which proved the existence of EVs in hP-MSC culture medium after ultracentrifugation. The internalization process further verifies the hypothesis that EVs can work as communication signals between MSCs and host cells. Furthermore, we also found the existence of EVs in the HCE culture medium, which confirms the existence and potential function of EVs in the communication between different types of cells.

After clarifying the presence of EVs and its ability to communicate between cells, we want to understand the role of EVs in tissue damage repair. Previous studies demonstrated that a rapid reestablishment of an intact epithelium in the injury area is critical for corneal wound healing [25]. This is a highly regulated process that requires the proliferation and migration of epithelial cells and stroma cell. Here, we show that hP-MSC-derived EVs cocultured with HCEs could improve epithelial cell proliferation and migration, as evidenced by the higher expression of Ki-67 and the scratch test in vitro. Moreover, corneal fluorescein sodium staining results show that hP-MSC-derived EVs improve corneal epithelial repair in an *in vivo* model of corneal alkali injury; the integration of epithelial layers is significantly increased.

In addition to HCE proliferation and migration, the angiogenesis and inflammation regulation also can influence the repair progress after injury [26]. The angiogenesis process of the cornea is often associated with an inflammatory and hypoxia response after injury of the cornea [27, 28]. During this repair process, several types of cells, stromal fibroblasts, and growth factors are involved in it [29, 30]. Therefore, many therapeutic options for these complications are target modifying VEGF and inflammatory cytokine level within the ocular surface to inhibit abnormal angiogenesis. Our study proved that EVs are involved in limiting the production of proinflammatory factors (TNF-*α*, IL-1*β*, IL-8, and NF-*κ*B) and caspase-8. In the meantime, EVs upregulate anti-inflammatory cytokine IL-10. EVs partially ameliorate the corneal histopathological changes induced by alkali injury. The blood vessel density of the cornea is much lower in the treatment group.

Although the upregulation of VEGF after cornea injury is associated with vision loss, it also plays a vital role in cellular homeostasis and tissue repair [31–33]. The efficacy of anti-VEGF treatments varies among patients and results in only partial vessel regression [34]. Long-term systemic administration of antiangiogenesis drugs coupled with several complications; these adverse effects draw concern that VEGF antagonism may not be completely effective or safe [35]. Hence, EVs could be a potential treatment strategy for corneal wound healing. Our study represents a novel method for treating corneal alkali burn by the administration of hP-MSC-derived EVs. Further studies are needed to clarify the exact mechanisms in this treatment method and the manipulation of EV compositions produced by hP-MSCs.

## 5. Conclusion

In conclusion, EVs derived from hP-MSCs are interesting potential therapeutic agents for improving corneal wound healing. It combines several mechanisms of the actions by stimulation of HCE migration and proliferation, inhibition of pathological angiogenesis, and modulation of MMP activity. Further studies are required to investigate the clinical benefit of MSC-derived EVs as a therapeutic agent for improving wound healing and reducing scar formation.

## Data Availability

All data generated and/or analyzed during this study are available from the corresponding author upon reasonable request.

## Supplementary Materials

Supplementary Materials Table S1: RT-PCR primer sequences (human). Table S2: RT-PCR primer sequences (mouse). Figure S1: flow cytometry analysis of phenotypic profiling of hP-MSCs.
