# Correction to “Mesenchymal Stem Cell‐Derived Extracellular Vesicles for Corneal Wound Repair”

**DOI:** 10.1155/sci/9798482

**Published:** 2026-07-22

H. Tao, X. Chen, H. Cao, et al., “Mesenchymal Stem Cell‐Derived Extracellular Vesicles for Corneal Wound Repair,” *Stem Cells International*, vol. 2019 (2019). https://doi.org/10.1155/2019/5738510.

In the article titled “Mesenchymal Stem Cell‐Derived Extracellular Vesicles for Corneal Wound Repair,” there was an error in Figure 6. More specifically, the description of the qRT‐PCR process in panel (b) and the assignment of the EV and PBS groups as calibrator groups were incorrect. These errors were introduced by the authors during manuscript preparation, and Figure 6 should be corrected as follows:

**Figure 6:**
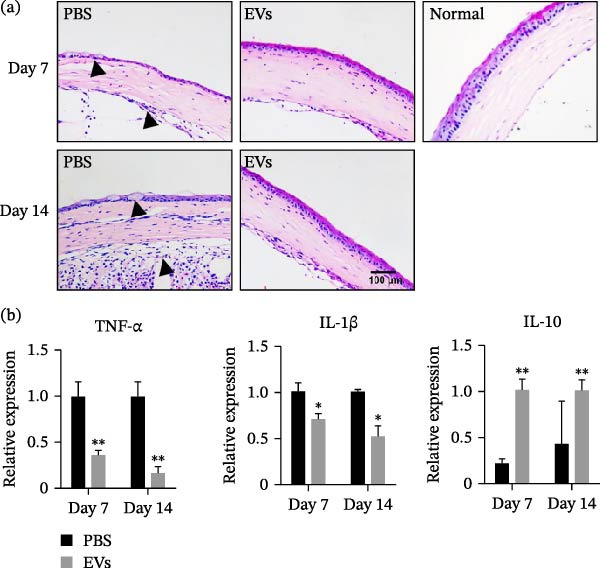
Enhanced therapeutic effect of EVs in the alkali burn model. (a) H&E staining of the cornea demonstrated stromal edema and inflammatory cell infiltration on Days 7 and 14 after injury in EV‐treated mice, compared with the cornea without injury. The arrowheads pointed to the disorganized fibers and phlyctenule in the epithelium layer. The degree of repair of the epithelial layer in the control group was significantly lower than that in the treatment group. (b) qRT‐PCR analysis of TNF‐α, IL‐1β, and IL‐10 mRNA in corneal tissue at Day 7 and Day 14 after alkali injury. Relative expression was calculated by the 2^−ΔΔCt^ method using GAPDH as the internal reference. To present upregulated and downregulated genes on a common *y*‐axis (0–1.5), the higher‐expressing group at each time point was used as the calibrator (set to a relative expression of 1): the PBS group for TNF‐α and IL‐1β, and the EVs group for IL‐10. Asterisks indicate statistical comparison of EVs versus PBS within the same time point (two‐tailed Student’s *t*‐test). Data are shown as mean ± SEM from triplicate assays.  ^∗^ *p* < 0.05,  ^∗∗^ *p* < 0.01.

We apologise for these errors.

