# Supplementary material for: Mesenchymal Stem Cell-Derived Extracellular Vesicles for Corneal Wound Repair
Source: Stem Cells Int. 2019 Dec 9;2019:5738510. doi: 10.1155/2019/5738510 (PMC6925772; doi:10.1155/2019/5738510)
Supplement: Supplementary Materials — Table S1: RT-PCR primer sequences (human). Table S2: RT-PCR primer sequences (mouse). Figure S1: flow cytometry analysis of phenotypic profiling of hP-MSCs. [file 5738510.f1.pdf]

Mesenchymal Stem Cell-derived Extracellular Vesicles with Chitosan Hydrogels for Corneal Wound Repair

Hongyan Tao, Xiaoniao Chen, Hongmei Cao, Lingyue Zheng, Qian Li, Kaiyue Zhang, Zhibo Han, Zhongchao Han, Zhikun Guo, Zongjin Li, Liqiang Wang

Supplementary Information:

Supplementary Table 1. RT-PCR primer sequences (human).

HUMAN		SEQUENCE (5' TO 3')
**IL-1β**	F	GTG GCA ATG AGG ATG ACT TGT TC
	R	TAG TGG TGG TCG GAG ATT CGT A
**IL-8**	F	CTG ATT TCT GCA GCT CTG TG
	R	GGG TGG AAA GGT TTG GAG TAT G
**IL-10**	F	GTG ATG CCC CAA GCT GAG A
	R	CAC GGC CTT GCT CTT GTT TT
**TNF-α**	F	CTG CTG CAC TTT GGA GTG AT
	R	AGA TGA TCT GAC TGC CTG GG
**CASPASE 3**	F	TGG TTC ATC CAG TCG CTT TG
	R	CAT TCT GTT GCC ACC TTT CG
**CASPASE 8**	F	CTG CTG GGG ATG GCC ACT GTG
	R	TCG CCT CGA GGA CAC GCT CTC
**MMP2**	F	CTT CAC TTT CCT GGG CAA CA

	R	CAA CTA CGA TGA TGA CCG CA
**VEGFA**	F	TGT CTA ATG CCC TGG AGC CT
	R	GTC ACA TCT GCA AGT ACG TTC G

Supplementary Table 2. RT-PCR primer sequences (mouse).

**MICE**		**SEQUENCE (5' TO 3')**
**GAPDH**	F	TTG TCT CCT GCG ACT TCA AC
	R	GTC ATA CCA GGA AAT GAG CTT G
**IL-6**	F	CAG CAA GAT GAT CCC AAT GA
	R	CTC TTG AGC TTG GTG ACA A
**TNF-α**	F	CAC GCT CTT CTG TCT ACT G
	R	GAA GAT GAT CTG AGT GTG AGG
**IL-1β**	F	GTA TGA CTC TAC CCA CGG CAA GT
	R	TTC CCG TTG ATG ACC AGC TT

Supplementary figure

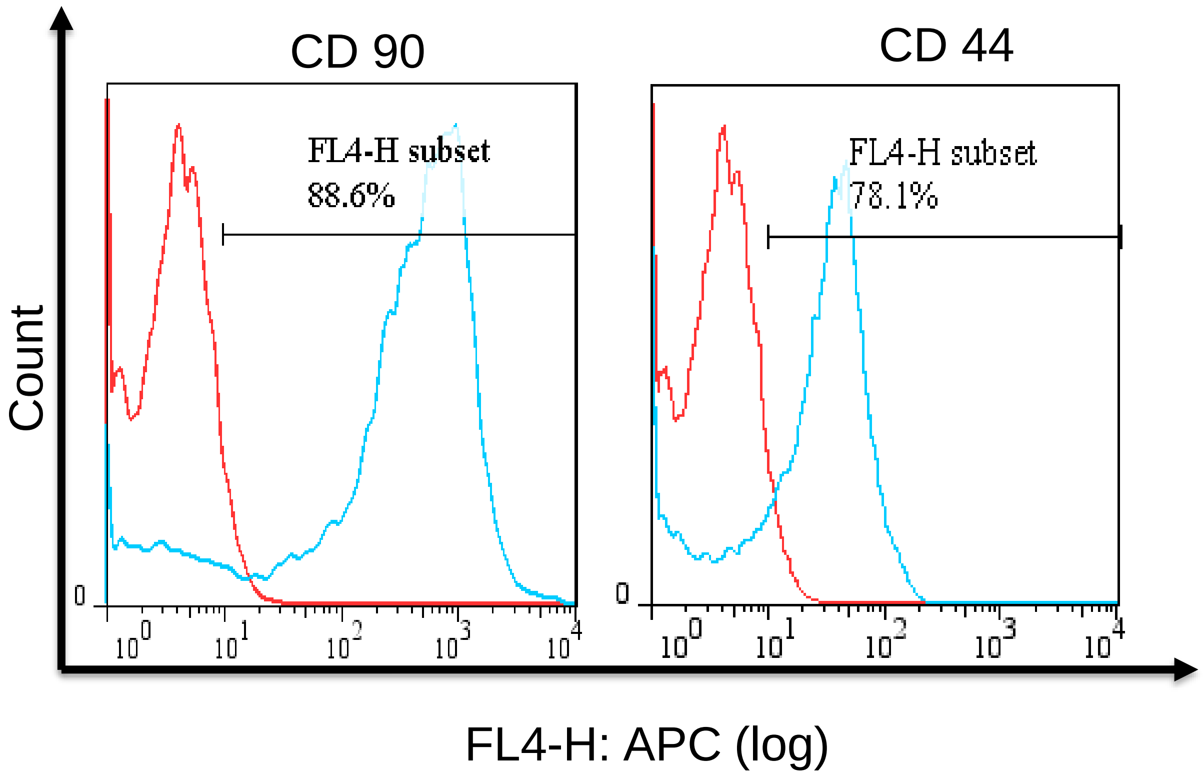

Supplementary Fig. 1: Flow cytometry analysis of phenotypic profiling of hP-MSCs.
